# Waldenström Macroglobulinemia Presenting With Hepatic Lesions: Case Report and Literature Review

**DOI:** 10.1155/crh/9908044

**Published:** 2026-06-09

**Authors:** Neta Sternbach, Shahd Bishara, Morie A. Gertz, Iuliana Vaxman

**Affiliations:** ^1^ Institute of Hematology, Davidoff Cancer Center, Rabin Medical Center Petah-Tikva, Petah-Tikva, Israel; ^2^ School of Medicine, Faculty of Medical and Health Sciences, Tel-Aviv University, Tel-Aviv, Israel, tau.ac.il; ^3^ Department of Pathology, Rabin Medical Center Petah-Tikva, Petah-Tikva, Israel; ^4^ Division of Hematology, Mayo Clinic, Rochester, Minnesota, USA, mayo.edu

**Keywords:** case report, extramedullary disease, granulomatous inflammation, hepatic lesions, Waldenström macroglobulinemia

## Abstract

Waldenström macroglobulinemia (WM) is a rare lymphoplasmacytic lymphoma characterized by bone marrow (BM) infiltration and monoclonal IgM secretion. Clinical manifestations most often reflect BM involvement, lymphadenopathy, or splenomegaly, while extramedullary disease is uncommon. Hepatic infiltration usually presents as diffuse hepatomegaly; focal hepatic lesions are exceedingly rare, with only two cases previously reported. We describe a 63‐year‐old woman with a history of celiac disease who presented with fatigue and pancytopenia. Laboratory testing showed elevated IgM with an IgM‐λ monoclonal protein. Imaging revealed multiple hypodense hepatic lesions, splenomegaly, and small‐volume lymphadenopathy. BM biopsy demonstrated lymphoplasmacytic lymphoma with *λ*‐restricted plasma cells, and molecular studies were negative for MYD88 L265P and CXCR4 mutations. Liver biopsy showed granulomatous inflammation with necrosis, accompanied by IgM‐positive, *λ*‐restricted B cells clonally related to the BM infiltrate, consistent with WM involvement. Sarcoidosis and infectious causes were excluded. The patient received six cycles of bendamustine and rituximab (BR), leading to a reduction in hepatic lesions and IgM levels, though treatment was complicated by persistent cytopenias. This case represents the first reported instance of WM presenting with focal hepatic lesions associated with IgM paraproteinemia and a sarcoid‐like granulomatous reaction. Recognition of such atypical presentations is essential, as they may mimic primary hepatic lymphoma, sarcoidosis, or infectious granulomatous disease. Comprehensive histopathological and molecular evaluation is critical for accurate diagnosis and appropriate therapy. Our report expands the spectrum of extramedullary WM and highlights the potential for favorable response to BR despite unusual presentation.

## 1. Case Report

The clinical course from presentation to diagnosis, treatment, and follow‐up is described chronologically.

A 63‐year‐old female with a past medical history of celiac disease (diagnosed based on positive celiac serology and supportive duodenal histology, managed with a gluten‐free diet alone) came to medical attention due to complaints of increased fatigue and cough. She denied fever, weight loss, or night sweats, as well as abdominal pain or discomfort. Her physical examination was unremarkable, with normal breath sounds and no lymphadenopathy or hepatosplenomegaly. She has no neuropathy and no signs of amyloidosis.

The peripheral blood test results showed pancytopenia, with a white blood cell count of 3 × 10^9^/L (normal range: 4.5–11 × 10^9^/L) and a neutrophil count of 60%, hemoglobin (Hb) concentration of 101 g/L, MCV of 96.6 fL, and platelet count of 148 × 10^9^/L (normal range: 150–450 × 10^9^/L). Biochemical analysis revealed increased levels of total serum globulin (40 g/L; normal range: 21–34 g/L) and lactate dehydrogenase (LDH) (293U/L; normal range: 135–214U/L). The levels of aspartate aminotransferase (AST), alanine aminotransferase (ALT), alkaline phosphatase (ALKP), total bilirubin, creatinine, and calcium were all normal. B_2_‐microglobulin levels were also normal. The level of serum IgM was 20.8 g/L (normal range: 0.4–2 g/L), and IgG and IgA levels were normal. An IgM‐λ monoclonal protein of 16.9 g/L was detected by immunoelectrophoresis with free light chain (FLC) *λ* of 11.3 g/L (normal range: 5.7–26.3 mg/L). C‐Reactive protein (CRP) levels were 0.8 mg/dL (normal range: up to 0.5 mg/dL), and erythrocyte sedimentation rate (ESR) was 57 mm/h. Vitamin B12, folate, iron and thyroid‐stimulating hormone (TSH) levels were normal. Cold agglutinin antibodies were detected in a titer of 1:64, and haptoglobin levels were within the normal range. A direct antiglobulin (Coombs) test was attempted but yielded inconclusive results and was not repeated.

Computed tomography (CT) scan revealed multiple hypodense lesions in the liver measuring up to 2 cm in size and up to 0.8 cm enlarged lymph nodes in the paratracheal, cervical, mediastinal, and retroperitoneal regions (Figure 1). The lesions in the liver were not demonstrated by ultrasound. Magnetic resonance imaging (MRI) performed 2 months after the CT scan demonstrated slight enlargement of the hepatic lesions, now measuring up to 2.4 cm, and in addition revealed splenomegaly of 15 cm (Figure 2) and high uptake foci in the BM with no lytic bone lesions.

**Figure FIGURE 1:**
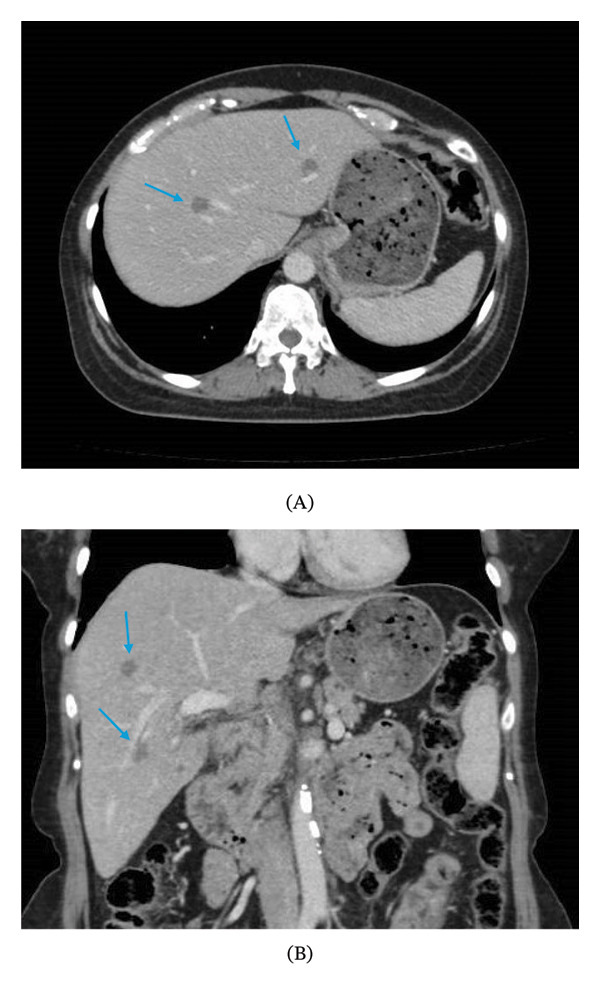
Computed tomography scan of the liver showing multiple hypodense lesions. (A) Axial section. (B) Coronal section.

**Figure FIGURE 2:**
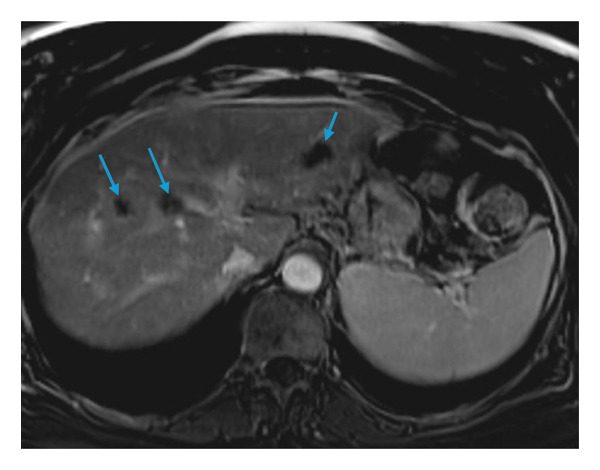
Magnetic resonance imaging of the liver showing multiple hepatic lesions.

The patient was evaluated by a pulmonologist for a persistent cough; sputum cultures repeatedly grew *Haemophilus influenzae*, and she was treated with azithromycin prophylaxis, with symptoms attributed to recurrent respiratory infections.

A BM biopsy indicated involvement by low‐grade B‐cell lymphoma, compatible with lymphoplasmacytic lymphoma. BM biopsy showed variable cellularity, between 5% and 70% overall. In the well‐preserved areas, an interstitial lymphocytic infiltrate was seen, mainly made up of small B‐lymphocytes comprising 15% of the overall cellularity (Figure 3). The proliferation index was low, with Ki67 of less than 1%. B‐Lymphocytes were positive for CD20 and CD19 and were negative for Cyclin D and LEF1. The vast majority of the B‐lymphocytes were also negative for CD23. The plasma cells accounted for 7% of cells in the BM and were positive for CD138 and lambda chain restricted (Figures 4A and B). MYD88 L256P mutation was not detected. CXCR4 was negative. Residual trilineage hematopoiesis was preserved, with orderly maturation of erythroid and myeloid lineages and no significant dysplastic features; megakaryocytes were present with unremarkable morphology. Liver biopsy specimens were involved by an extensive granulomatous process with areas of necrosis surrounded by macrophages and giant cells. Around the areas of necrosis, a lymphoplasmocytic process is seen, consisting of small lymphocytes (some are B and some are T cells). B‐cells are arranged in aggregates surrounding the ducts within the liver parenchyma (Figure 5). The clonal rearrangement of the immunoglobulin heavy chain (IGH) gene was evaluated using polymerase chain reaction (PCR) amplification. The analysis showed these B cells were derived from the same B‐cell clone (Table 1). The lymphocytes are accompanied by a few mature plasma cells and are positive for MUM1, monoclonal positivity for lambda light chain, and positive for IgM. The liver was minimally involved by the same process seen in the BM. *Mycobacterium tuberculosis* complex test by PCR, IGG4 staining was negative, and serum angiotensin–converting enzyme (ACE) was mildly elevated.

**Figure FIGURE 3:**
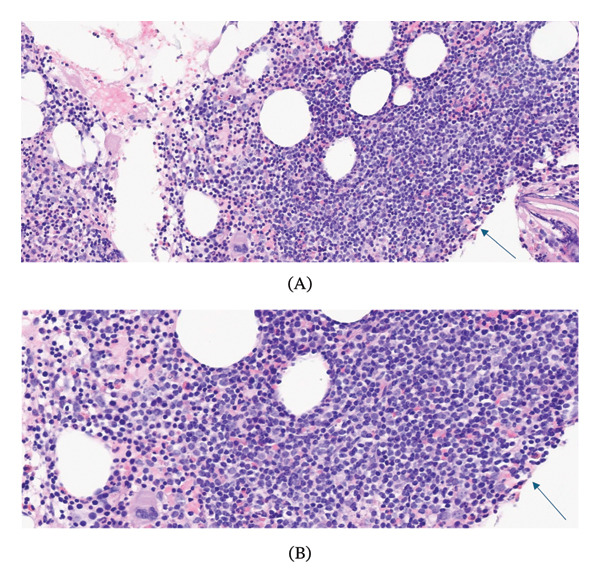
H&E staining of BM biopsy showing interstitial lymphocytic infiltrates. (A) 20x magnification. (B) 40x magnification.

**Figure FIGURE 4:**
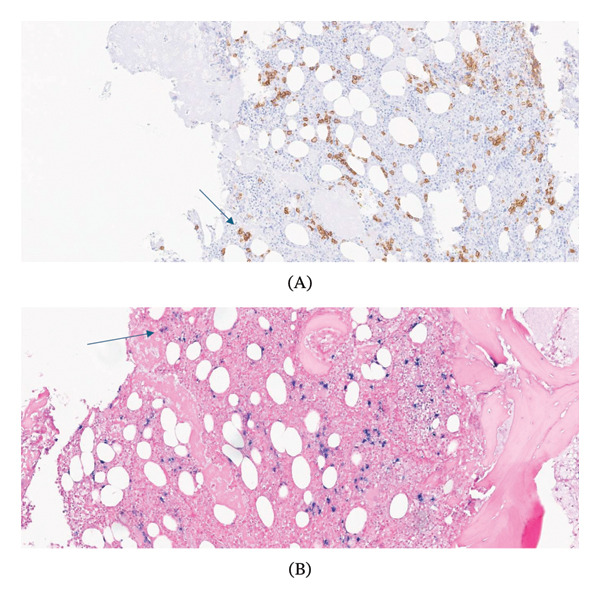
Bone marrow biopsy. (A) CD138 immunohistochemical stain. (B) Lambda chain immunohistochemical stain.

**Figure FIGURE 5:**
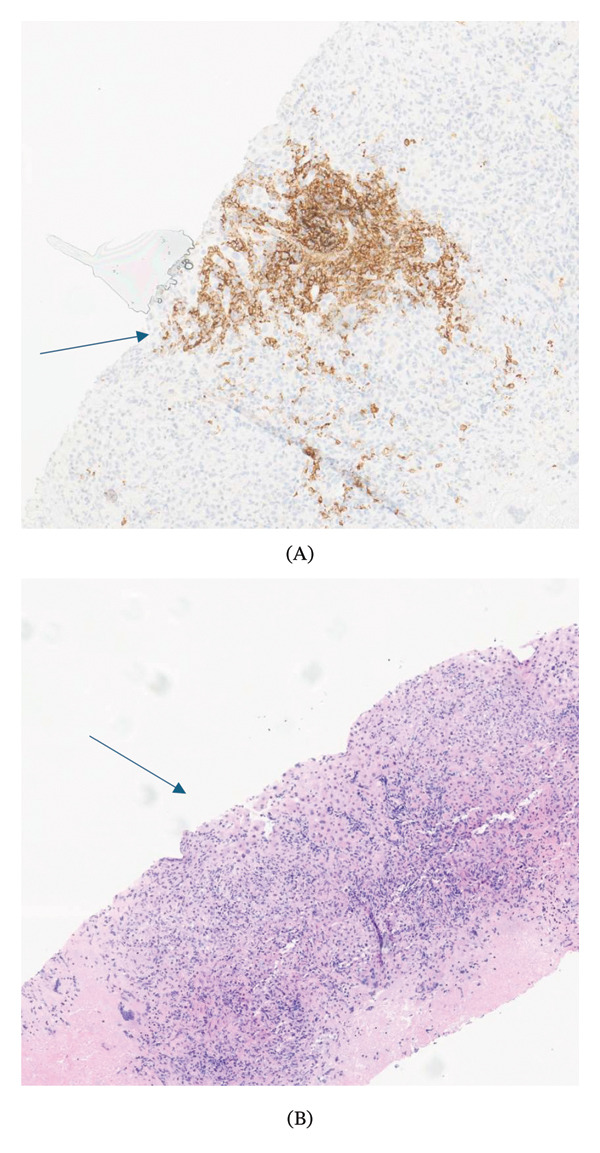
Liver biopsy. (A) CD20 immunohistochemical stain. (B) H&E staining of liver biopsy showing interstitial lymphocytic infiltrates.

**Table TABLE 1:** PCR analysis for B‐cell clonality in liver biopsy showing monoclonality.

Gene	Primers	Test results
IGH	VH‐FR1‐JH	Peak (334 bp)
IGH	VH‐FR2‐JH	Peak (269 bp)
IGH	VH‐FR3‐JH	Peak (127 bp)

These findings established a diagnosis of Waldenström macroglobulinemia (WM) with liver infiltration, low risk according to the International Prognostic Scoring System (IPSS).

The patient received treatment with six cycles of BR (bendamustine 90 mg/m^2^ for 2 days, rituximab), with a decrease in the number and size of hepatic lesions, with low F‐fluorodeoxyglucose (FDG) uptake as evident on an interim positron emission tomography (PET) scan (performed 1 month after completing the fourth cycle). The fifth treatment was delayed due to infectious complications and prolonged Grade 3/4 neutropenia. Three months after the end of treatment, total IgM levels were 974 mg/dL, and the patient is still cytopenic with Hb of 11.4 g/dL, platelets of 80 × 10^9^/L, and WBC of 2.2 × 10^9^/L, of which 67% were neutrophils. Follow‐up imaging demonstrated a favorable hepatic response, with a reduction in lesion size and metabolic activity. However, splenomegaly persisted without a reduction in size, and a new small hypodense splenic lesion without FDG uptake was noted.

## 2. Discussion

WM is a distinct clinicopathologic entity characterized by lymphoplasmacytic lymphocytes infiltrating the bone marrow (BM) and an IgM monoclonal gammopathy in the blood. Upon diagnosis, approximately 25% of individuals with WM exhibit no noticeable symptoms [1]. Typically, initial symptoms, which can take years to manifest, are nonspecific and include fatigue, malaise, weight loss, and fever. As the disease progresses, patients may develop symptoms related to the infiltration of organs and tissues and the IgM paraprotein. Extramedullary hematopoietic tissue infiltration is seen in 25% of cases and can cause lymphadenopathy, hepatomegaly, and splenomegaly [2]. Tissue infiltration of other organs by neoplastic cells is rare and can involve various organs and tissues. Hepatomegaly is usually of a mild to moderate degree. Focal hepatic lesions are extremely rare, and liver function test results are typically normal [3].

In our patient, cytopenias were likely multifactorial, reflecting a combination of BM involvement, splenomegaly, and possible disease‐related inflammatory effects.

In our literature search, we found only two case reports that describe WM presenting as hepatic focal lesions. Oshima et al. reported a case of a 70‐year‐old female patient with a diagnosis of lymphoplasmacytic lymphoma that presented as a hepatic bulky mass in the presence of IgG‐kappa paraproteinemia and in the absence of a MYD88 L265P mutation. The patients progressed on the first line of treatment and eventually achieved a partial response with the bendamustine and rituximab (BR) protocol [4]. Another case from 1995 was of primary lymphoma of the liver, exhibiting the immunohistological features of a B‐cell low‐grade lymphoma of the lymphoplasmacytic type, successfully treated by major liver resection [5]. Interestingly, both cases were associated with an IgG‐kappa monoclonal protein. Our patient is the first case report of WM with a liver mass that is related to IgM.

In a study from 2014 that reported the characteristics of WM patients with extramedullary disease, among the 985 WM patients, 43 (4.4%) had evidence of extramedullary involvement, excluding hepatosplenomegaly or lymphadenopathy (EMD). Among the 43 WM patients identified with EMD, 9 (21%) patients presented with involvement at WM diagnosis, and the other 34 (79%) presented with EMD posttherapy for WM. The most frequent EMD sites involved were pulmonary (30%), soft tissue (21%), cerebrospinal fluid (23%), renal (8%), and bone (9%). Only one patient had liver involvement, identified at diagnosis. The median overall survival at 10 years for patients with extramedullary WM was comparable to that of patients with WM when all IPSS risk factors were considered. This suggests that extramedullary WM remains potentially treatable and does not confer a poorer prognosis [6].

Hepatic lymphoma involvement can be classified into primary (confined to the liver and hilar lymph nodes without distant spread) and secondary (metastatic) forms. Secondary hepatic lymphoma is seen in nearly half of patients with gastrointestinal involvement, which is the most common extranodal site in non‐Hodgkin lymphoma (NHL). In contrast, primary hepatic lymphoma is rare, accounting for less than 1% of all NHLs [7]. The most common presentation of primary hepatic lymphoma is a solitary heterogeneous mass, while multiple lesions occur in fewer than 33% of cases [8]. Secondary hepatic lymphoma typically manifests as diffuse infiltration or multifocal homogeneous lesions alongside extrahepatic disease.

Noncaseating granulomas are observed in various pathological conditions. Hepatic granulomas associated with celiac disease were also considered; however, this was considered unlikely given the presence of a clonal IgM‐positive, lambda‐restricted lymphoplasmacytic infiltrate in the liver, consistent with WM involvement, and the absence of active celiac‐related systemic inflammation. Sarcoidosis, a multisystem inflammatory disorder, is a prime example of a condition that forms noncaseating granulomas, primarily affecting the lungs and lymph nodes. Although rare, the association between noncaseating granulomas and WM has been reported in at least four patients. In all cases, there has been prominent lung involvement with respiratory symptoms and hilar lymphadenopathy. In two cases, pathological findings showed simultaneous involvement of atypical lymphoplasmacytic cells together with noncaseating granulomas from hilar lymph node biopsies. In both cases, enlarged lymph nodes regressed in response to anti‐WM‐directed therapy. These two patients were not thought to meet the diagnosis of sarcoidosis [9, 10]. In a third case, the patient developed WM 7 years after the diagnosis of pulmonary and cutaneous sarcoidosis was made, and WM‐directed therapy was effective against both diseases [11]. A patient who developed WM involving the bone marrow three years after being diagnosed with pulmonary sarcoidosis, which had been successfully treated and resolved. No recurrence of sarcoidosis was observed at the time of WM diagnosis [12]. The exact relationship between sarcoidosis and WM is not well understood. However, it is believed that immune dysregulation and chronic inflammation may play a role in their co‐occurrence. Both conditions involve abnormalities in the immune system, which could contribute to their association. The possibility of sarcoidosis was considered in our patient, but it was ultimately ruled out since the patient had only mildly elevated levels of serum ACE, normal calcium levels, absence of granulomas on BM biopsy, and the lack of respiratory manifestations, which are highly prevalent in sarcoidosis. Noncaseating granulomas are also recognized in other hematologic malignancies, both Hodgkin and NHL [13]. Based on two studies, the incidence of sarcoid‐like granulomas and NHL occurring simultaneously at the same site is 3.6% [14, 15]. Worth noting are two sites of propensity to this phenomenon, including the skin and the lungs; 50% of low‐grade pulmonary lymphomas are associated with granulomas embedded in the lymphoid mass. The sarcoid‐like granulomas are usually not associated with clinical evidence of sarcoidosis. In cases in which both diagnoses are made in the same patient, each diagnosis must be independently confirmed by biopsy specimens from separate anatomic sites, alongside appropriate clinical, radiographic, and biochemical features for each disease; sarcoidosis, specifically, should demonstrate multisystem involvement.

In conclusion, we reported the first case of WM presenting as hepatic focal lesions with pathological findings of sarcoid‐like reaction.

## Author Contributions

NS and IV conceived the study. NS collected the clinical data and drafted the manuscript. SB contributed to the pathological analysis. MAG and IV critically revised the manuscript.

## Funding

No funding was received for this study.

## Disclosure

All authors have read and approved the final version of the manuscript. **Neta Sternbach** had full access to all of the data in this study and takes complete responsibility for the integrity of the data and the accuracy of the data analysis.

## Ethics Statement

Ethical approval was not required for this case report in accordance with institutional policies.

## Consent

Written informed consent was obtained from the patient for publication of this case report and accompanying images.

## Conflicts of Interest

The authors declare no conflicts of interest.

## Data Availability

The original data are available from the corresponding author.
